# Early successional dynamics of ground beetles (Coleoptera, Carabidae) in the tropical dry forest ecosystem in Colombia

**DOI:** 10.3897/zookeys.1044.59475

**Published:** 2021-06-16

**Authors:** Gloria Maria Ariza, Jorge Jácome, Héctor Eduardo Esquivel, D. Johan Kotze

**Affiliations:** 1 Departamento de Biología, Unidad de Ecología y Sistemática (UNESIS), Pontificia Universidad Javeriana, Bogotá, Colombia; 2 Departamento de Biología, Unidad de Ecología y Sistemática (UNESIS), Pontificia Universidad Javeriana, Bogotá, Colombia; 3 GIBDET Research group, Universidad del Tolima, Ibagué, Colombia; 4 Faculty of Biological and Environmental Sciences, Ecosystems and Environment Research Programme, University of Helsinki, Niemenkatu 73, 15140, Lahti, Finland

**Keywords:** Climatic variation, ENSO, environmental variables, natural recovery, seasonality

## Abstract

Little is known about the successional dynamics of insects in the highly threatened tropical dry forest (TDF) ecosystem. For the first time, we studied the response of carabid beetles to vegetal succession and seasonality in this ecosystem in Colombia. Carabid beetles were collected from three TDF habitat types in two regions in Colombia: initial successional state (pasture), early succession, and intermediate succession (forest). The surveys were performed monthly for 13 months in one of the regions (Armero) and during two months, one in the dry and one in the wet season, in the other region (Cambao). A set of environmental variables were recorded per month at each site. Twenty-four carabid beetle species were collected during the study. *Calosoma alternans* and *Megacephala affinis* were the most abundant species, while most species were of low abundance. Forest and pasture beetle assemblages were distinct, while the early succession assemblage overlapped with these assemblages. Canopy cover, litter depth, and soil and air temperatures were important in structuring the assemblages. Even though seasonality did not affect the carabid beetle assemblage, individual species responded positively to the wet season. It is shown that early successional areas in TDF could potentially act as habitat corridors for species to recolonize forest areas, since these successional areas host a number of species that inhabit forests and pastures. Climatic variation, like the El Niño episode during this study, appears to affect the carabid beetle assemblage negatively, exasperating concerns of this already threatened tropical ecosystem.

## Introduction

Tropical dry forest (TDF) is considered the most threatened tropical ecosystem in South America and Africa (Janzen 1988; Miles et al. 2006) and is simultaneously one of the lesser-known ecologically (Sánchez-Azofeifa et al. 2005). Recent studies have shown that TDF has high levels of beta diversity and endemism, which could be lost if inappropriate conservation strategies are applied (Banda-R et al. 2016). In the Americas, Colombia hosts extensive dry forest areas (Miles et al. 2006). However, with high anthropogenic pressure, these forests are historically characterized by high levels of fragmentation (Etter et al. 2008), which in turn results in a reduction in habitat connectivity (Balzotti et al. 2020), ecosystem functions and services (Balvanera et al. 2011). Today, only 8% of the original TDF in Colombia remains (Pizano et al. 2016). An example is TDF in the Valley of the Magdalena River in Colombia, which has experienced intensive agriculture resulting in a heterogeneous landscape (Fernández-Méndez et al. 2014), with most forest patches reduced to less than 30 ha in size (Pizano et al. 2016). This landscape mosaic is characterized by areas at various stages of succession, similar to other countries (Quesada et al. 2009), which generate potential sources for spontaneous succession (Prach 2003). Spontaneous succession is considered a cheap and rapid recovery tool (Prach and Pyšek 2001), which in the case of TDF could improve connectivity, at least for poorly dispersing insects species (Aparício et al. 2018). As such, the application of conservation strategies and ecological restoration is of paramount importance (Vargas and Ramírez 2014), especially since TDF succession is slow compared to wet tropical forest (Murphy and Lugo 1986), because water is a limiting factor when it comes to recovery (Fajardo et al. 2013).

Knowledge on succession, defined as species turnover with time (Walker and del Moral 2003), is necessary to understand changes experienced by communities due to anthropogenic disturbances as well as the activities necessary for their recovery (Prach 2003; Prach and Walker 2011). Since insects are a major component of terrestrial ecosystems (Samways 1994; Schowalter 2006; Scudder 2009), knowledge on how they respond to succession is paramount. Yet, tropical insects do not show consistent patterns during succession and appear to be highly dynamic; their response to succession depends on the community variable evaluated (species richness, diversity or abundance), the region, and type of disturbance (e.g., Hilt et al. 2006; Neves et al. 2010a; Hernández et al. 2014; Nyafwono et al. 2014). For TDF insects, successional pathways are also difficult to predict, since changes are related to seasonal variability (Neves et al. 2010a). Seasonal fluctuations in insects in TDF are well document (e.g., Noguera-Martínez et al. 2007; Pérez and Zaragoza 2016; Corona-López et al. 2017; Noguera et al. 2018), however, peaks in species richness are not clear when successional stages are included (e.g., Neves et al. 2010b). Additionally, taxa occupying different forest strata respond differently to succession; e.g., dung beetles and hypogaeic ant richness change with succession (Neves et al. 2010a; Marques et al. 2017), while arboreal and epigaeic ant do not (Neves et al. 2010b; Marques et al. 2017). The mechanisms of these differences are not completely understood, but may be related to specific resource use and abiotic requirements (Neves et al. 2014).

Carabidae is a large coleopteran family (ca. 34,000 species) (Bousquet 2010), extensively distributed and with high abundance (Larochelle and Larivière 2003), making them a prevalent model organism, especially in the temperate region (Koivula 2011). The well-documented information about its taxonomy and biology, and its response to environmental change helped in its wide used as bioindicators (Koivula 2011; Kotze et al. 2011). However, in the Neotropics, little is known about this group (Lucky et al. 2002; Rainio and Niemelä 2003; Martínez 2005; Maveety et al. 2011). One considerable hurdle is taxonomy with few identification keys and a great number of undescribed species (Lucky et al. 2002; Maveety et al. 2011; Erwin et al. 2015). Knowledge on carabid beetles in TDF is scarce, with most studies dealing with assemblage characterization (Arenas et al. 2013; Uribe and Vallejo 2013; Arenas and Ulloa-Chacón 2016). Baseline information on how carabid beetle assemblages respond to succession, and their seasonal dynamics in TDF, is urgently needed both from an ecological and conservation perspective. The present study is the first to investigate these issues in this highly threatened ecosystem.

The overall aims of our study were to investigate carabid beetle assemblage changes during early succession in TDF in Colombia, and their response to environmental variables along this successional process. Furthermore, given the strong seasonality experienced in this ecosystem, and that the data were collected during an El Niño event (Varotsos et al. 2016; Whitfield et al. 2019), we evaluate the response of this group to wet and dry periods. The El Niño/Southern Oscillation (ENSO) is a periodic climatic event that affects inter-annual rainfall regimes. In Colombia, it consists of a dry episode with a precipitation deficit and raising air temperatures (Poveda et al. 2000). It can produce severe droughts as has happened in Colombian TDF during the study period (Montealegre 2014).

We hypothesize that the carabid beetle assemblage in early successional TDF is speciose with high abundance compared to forest (e.g., Magura et al. 2015; Barber et al. 2017), due to the arrival of open-habitat and habitat generalist species (similar to temperate ground beetles) (e.g., Nagy et al. 2016). If the majority of carabid species of the TDF matrix are polyphagous predators (Lövei and Sunderland 1996) and habitat generalists (Rainio and Niemelä 2003) (as in other ecosystems), they will benefit from exploiting resources in the initial stages of TDF recovery, where resources are heterogeneous (Lebrija-Trejos et al. 2009). We also expect carabid beetles to respond to architectural attributes of the habitat, like vegetation cover and leaf litter (e.g., Molnár et al. 2001), since cover and litter influence environmental conditions (Facelli and Pickett 1991; Lebrija-Trejos et al. 2011), and are considered important in structuring carabid assemblages (Koivula et al. 1999; Antvogel and Bonn 2001). However, we expect that soil humidity will be a major factor that affects ground beetles in this ecosystem, because i) it has been showed to influence carabid assemblages (Niemelä et al. 1992; Kaizuka and Iwasa 2015; Fidan and Sirin 2016), ii) water is a structuring and limiting factor of dry forest (Maass and Burgos 2011), and iii) dry habitat carabid beetles synchronize their life cycle to optimal soil humidity conditions (Paarmann 1979; Paarmann et al. 1986). Other insects in TDF have shown to be influenced by soil/litter humidity, which are linked to precipitation (García et al. 2001; Rangel-Acosta and Martínez-Hernández 2017).

## Materials and methods

### Study areas

**Figure 1.**
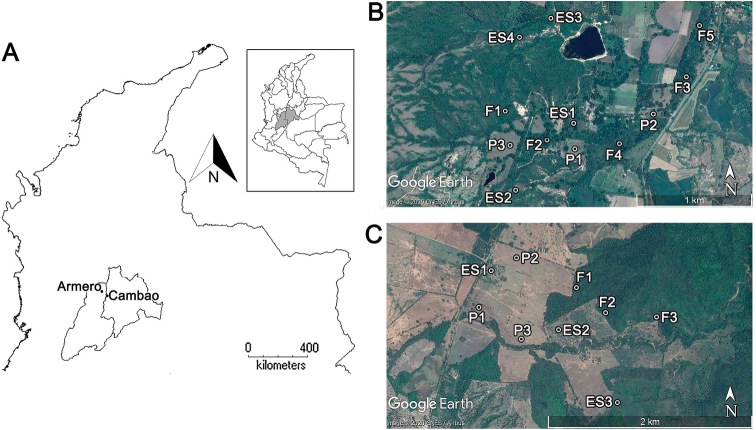
Geographic location of the study sites **A** the location of Armero and Cambao in Colombia **B** Armero **C** Cambao. Abbreviations: F = forest, ES = early succession, P = pasture. Maps courtesy of DIVA-GIS 7.5 and Google Earth Image 2020. For more details, see the online map at https://www.google.com/maps/d/u/3/edit?mid=1le-kQOQFh8RumUibWP3D8ghtxVvGM-eF&usp=sharing

The study was performed in the tropical dry forest biome in the Valley of the Magdalena River region (Colombia), in the municipalities of Armero-Tolima (305 m a.s.l.) and Cambao-Cundinamarca (294 m a.s.l.), both of which consist of a matrix of forest, pasture, and crops (Fig. 1). The average annual temperature is 27.4 °C in Armero and 28.5 °C in Cambao. Annual precipitation is 746.7 mm and 744.8 mm, respectively. This biome is characterized by two periods of marked drought in December–March and July–September.

The disturbance history of dry forest in this region is highly variable, due to agriculture and cattle ranching (clear-cutting), the use of timber trees (selective cutting) and a volcanic eruption in 1985 (Fernández-Méndez et al. 2014; Esquivel et al. 2016). In Armero the forest patches are of two types: forests that have never been clear-cut but experience selective cutting (see F1–2 in Fig. 1B), and forests with 32 years of primary succession (F3–5 in Fig. 1B). In Cambao (Fig. 1C), forests are areas with 15 years of secondary succession. Using a floristic characterization (Suppl. material 1: Table S1), these forest areas were classified as being at an intermediate stage of succession, defined as arbustive areas with between 10 and 50 years of succession (Nassar et al. 2008).

We collected carabid beetles in three successional stages in Armero and Cambao: pasture as an initial point, early succession (3–7 years of succession), and forest (intermediate successional stage). Each habitat type was replicated three times per area except for the forest and early successional sites in Armero, which had five and four replicates, respectively. This resulted in 12 sites at Armero and 9 sites at Cambao (Fig. 1). The minimum distance between sites within a study area was 240 m and the maximum distance was 2.2 km, while Armero and Cambao are 25 km apart.

### Carabid beetle sampling

Epigaeic ground beetles were collected using 300 ml transparent pitfall traps (7 cm Ø), which were filled three-quarters of the way with a solution of water and a few drops of detergent. The surveys were performed on a monthly basis (the traps were active for three days per month) for 13 months from June 2015 to June 2016 in Armero. Due to financial constraints, surveys in Cambao were only performed during two months, one in the dry season (December 2015) and one in the wet season (May 2016).

Ten traps were installed at each site along a transect of 100 m, with traps 10 m apart. Each transect was at least 20 m from the edge of the site to minimize edge effects, however, average distance from the edge was 140 m. The catch of the ten traps per site was pooled per visit. A trap was considered “lost” if it disappeared, was flooded, or dried in its entirety: 10.4% of the traps were lost in Armero and 5% in Cambao. Losses were considered in the statistical analyses (see below). Adult carabid beetles (including the subfamily Cicindelinae, see Bousquet 2012) were identified to genus level using taxonomic keys of the region from Martínez (2005) and to species level using taxonomic keys and/or original descriptions from Dejean (1829, 1831), Putzeys (1846, 1866), Reichardt (1967), Ball et al. (1991), Ball and Shpeley (2002, 2009), Vitolo (2004), Will (2005), and Bruschi (2010). However, given the scarcity of taxonomic keys for the Neotropics, some of the identifications should be confirmed. Voucher specimens are deposited in the Entomological Museum of the Universidad del Tolima, Colombia (MENT-UT) (Suppl. material 2: Table S2).

### Environmental variables

A set of environmental variables, including soil and air humidity and temperature, leaf litter depth and canopy cover were recorded per month at each site. Soil measurements (humidity and temperature) were taken using an Em50 Decagon digital data logger, which was installed in the vicinity of each transect and programmed to take measurements at 30 min intervals during three hours at midday (between 11:00 and 14:00) and then averaged. Air moisture and temperature were measured 2 cm above ground in the middle of the transect, using an Extech Thermohygrometer. Both soil and air variables were registered once per month in each site during the survey event.

Canopy cover (as a percentage) was calculated with a spherical crown densitometer at each pitfall trap (Lemmon 1956); the 10 measures per transect were averaged. The same was done with leaf litter depth, which was determined using the Kostel-Hughes et al. (1998) method. This method consists of inserting a wooden stick (3 mm in diameter) into the litter until it touched the humus layer. Four litter depth measures were taken per pitfall trap, two at 25 cm distance from the trap and two at 50 cm from the trap (Suppl. material 3: Table S3).

### Statistical analyses

Data were analyzed at the individual species and assemblage levels to determine how ground beetles respond to habitat type (forest, early succession, and pasture), environmental variables and seasonality. Analyses were performed on two datasets; Armero (13 months of data), and Armero and Cambao combined (two months of data collected per locality, December 2015 and May 2016).

For both datasets, species richness of each habitat type was calculated using sample-size-based and coverage-based rarefaction/extrapolation curves with Hill numbers (q = 0) (Chao et al. 2014), using the iNEXT package (Hsieh et al. 2016) in R (R Core Team 2020). This approach estimates richness for standardized samples (number of individuals) using a common sample size (114 individuals for Armero and 321 for Armero and Cambao combined) and sample completeness (0.97 for Armero and 0.99 for Armero and Cambao combined). The 95% confidence intervals were calculated using bootstrapping (200 bootstrapped samples).

Non-metric multidimensional scaling (NMDS) was used to display the response of the carabid beetle assemblage at Armero to habitat type, seasonality, and the measured environmental variables. The analysis was run with the vegan package (Oksanen 2015) in R, using the Horn measure as similarity index (Jost et al. 2011). The envfit function in vegan was used to evaluate the significance of seasonality and environmental variables in explaining the structure of the beetle assemblage. A permutational multivariate analysis of variance (PERMANOVA) test was performed, using the adonis2 function and the Horn similarity measure in vegan, to evaluate whether the carabid beetle assemblages were significantly different between the three habitat types. The beetle catch was standardized to 60 traps per season (wet or dry) per site to take into account lost traps.

Generalized linear models (GLMs) were run in R to relate habitat type (as a factor), environmental variables and seasonality to abundantly collected species in Armero (13 months of data). The most abundant species were analyzed individually (with more than 100 individuals collected), while species of lower abundances were grouped together; models with species of fewer than 100 individuals collected were unstable with unrealistic coefficients and standard errors. Species collected in Armero were analyzed using the glm function in the lme4 package, with the response variable (active density, hereafter referred to as abundance) modelled following a quasi-Poisson distribution (see Ver Hoef and Boveng 2007). The following variables were included in the models: 1) logged trap number as an offset term to account for trap losses (Kotze et al. 2012), 2) habitat type as a factor, 3) season as a two-level factor (dry and wet), and 4) environmental variables (soil and air humidity and temperature, leaf litter depth and canopy cover). To minimize collinearity between environmental variables, a correlation was run using the corrplot package in R. Canopy cover and air humidity and temperature were removed because they correlated strongly with soil temperature (r = -0.72, p < 0.001; r = -0.62, p = 0.004; r = 0.83, p < 0.001, respectively). Then a VIF (variance inflation factor) was run using the car package in R (Fox et al. 2016) to test collinearity of the environmental variables in the final models. Litter depth was removed from the *C. alternans* model (VIF = 10.94), and soil humidity was removed from the “rest of the species” model (VIF = 5.82).

Generalized linear mixed models (GLMMs) were run in R to relate habitat type (as a factor), environmental variables and seasonality to abundantly collected carabid beetle species for Armero and Cambao combined (two months of data per locality). The most abundant species were analyzed individually (with more than 39 individuals collected), while species of lower abundances were grouped together. The glmer function in the lme4 package (Bates et al. 2015) was used to analyze the Armero and Cambao combined dataset. Abundance data (per species) were modelled following a Poisson distribution (see O’Hara and Kotze 2010) and an observation-level random effect was added to deal with possible overdispersion (Harrison 2014). The following fixed effects were included in the GLMM models: 1) logged trap number as an offset term to account for trap losses, 2) habitat type as a factor, 3) season as a two-level factor (dry and wet), and 4) environmental variables (soil and air humidity and temperature, leaf litter depth and canopy cover). Study area (Armero and Cambao) was added as a random term to account for locality effects. Air (r = -0.6, p = 0.001) and soil humidity (r = -0.57, p = 0.002) and air temperature (r = 0.51, p = 0.009) were removed because they correlated with soil temperature. For *C. alternans* and the “rest of the species” models, canopy cover was removed from the final models (VIF = 5.26, 7.72, respectively). We performed model selection on both GLMs and GLMMs by removing non-significant environmental variable terms one at a time, but habitat type was retained even if statistically insignificant since it was part of the main design. Model validation was performed using the k-fold cross-validation procedure in the R library caret (Kassambara 2018). The predict function was used to predict the number of individuals from the final models. Finally, using the package multcomp and the function glht in R, a Tukey’s HSD post‐hoc test was performed to identify significant differences between habitat type categories.

## Results

### Distribution of species among habitat types

Eighteen carabid beetle species (182 individuals) were collected in Armero and ten species (355 individuals) in Cambao (Table 1). In Armero, seven species were collected from forest, with *Anaulacus piceolus* (Chaudoir) exclusively from this habitat type. In pasture, nine species were collected, with *Apenes* sp. 1, *Barysomus hoepfneri* Dejean, and *Selenophorus parvus* Darlington occurring only in this habitat type. Most species were collected from the early succession habitat type (13 species), also with four exclusive species (*Apenes* sp. 2, *Pelecium laevigatum* Guérin-Méneville, *Stolonis notula* Motschulsky, and *Stolonis parvulus* (Straneo)). However, most exclusive species in these habitat types are singletons, and their habitat preference should be considered with caution. *Calosoma alternans* (Fabricius) and *Megacephala affinis* Dejean were the most abundantly collected species (113 and 21 individuals respectively), *C. alternans* occurring in all habitat types while *M. affinis* was not present in forest.

**Table 1.** Number of individuals of all carabid beetle species collected in each habitat type at Armero and Cambao, Colombia. The season column represents the season during which the species was collected; w = wet, d = dry; capital letter represents the season with the most abundant catch. Abbreviations: F = forest, ES = early succession, P = pasture.

Species	Habitat type						Total	Season
	F		ES		P			
	w	d	w	d	w	d		
**Armero**								
*Anaulacus piceolus* (Chaudoir, 1876)		1					1	d
*Apenes prasinus* Ball & Shpeley, 1992	2	1	1	1			5	dW
*Apenes* sp. 1					1		1	w
*Apenes* sp. 2				1			1	d
*Athrostictus chlaenioides* Dejean, 1829	1				2		3	w
*Athrostictus paganus* (Dejean, 1831)				1	1		2	dw
*Barysomus hoepfneri* Dejean, 1829					1	2	3	Dw
*Calosoma alternans* (Fabricius, 1792)	8	5	17	3	75	5	113	dW
*Clivina* sp.	1			2			3	Dw
*Enceladus gigas* Bonelli, 1813	2		2	4			8	dw
*Galerita* sp.	4	1		1			6	dW
*Megacephala affinis* Dejean, 1825			3	1	14	3	21	dW
*Meotachys* sp.				1		1	2	d
*Pelecium laevigatum* Guérin-Méneville, 1843			1				1	w
*Selenophorus parvus* Darlington, 1934					2	2	4	dw
*Stolonis notula* Motschulsky, 1866			1				1	w
*Stolonis parvulus* (Straneo, 1951)				1			1	d
*Tetragonoderus* sp.			1		2	3	6	dw
Total number of individuals	**18**	**8**	**26**	**16**	**98**	**16**	**182**	
Total number of species	**7**		**13**		**9**		**18**	
**Cambao**								
*Apenes* sp. 3						3	3	d
*Apenes morio* (Dejean, 1825)		1					1	d
*Calosoma alternans* (Fabricius, 1792)	1		15	4	273	6	299	dW
*Megacephala affinis* Dejean, 1825	1	1	7	8	1	2	20	Dw
*Megacephala cribrata* Steinheil, 1875			10	1	3	2	16	dW
*Selenophorus parvus* Darlington, 1934	1		1				2	w
*Selenophorus woodruffi* Ball & Shpeley, 1992			1	1	4	3	9	dW
*Selenophorus clypealis* Ball & Shpeley, 1992					2		2	w
*Stenomorphus angustatus* Dejean, 1831				2			2	d
*Tetragonoderus* sp.						1	1	d
**Total number of individuals**	**3**	**2**	**34**	**16**	**283**	**17**	**355**	
**Total number of species**	**4**		**6**		**7**		**10**	

In Cambao, four species were collected from forest, with *Apenes morio* (Dejean) exclusively so. Early succession and pasture had similar numbers of species (six and seven). *Stenomorphus angustatus* Dejean was collected exclusively from the early succession habitat type, while pasture had three exclusive species *Apenes* sp. 3, *Selenophorus clypealis* Ball & Shpeley, and *Tetragonoderus* sp. *Calosoma alternans* and *M. affinis* were the most abundantly collected species (299 and 20 individuals respectively), both occurring in all habitat types and in both localities (Armero and Cambao). *Megacephala affinis* was collected abundantly in pasture in Armero, but in the early succession habitat in Cambao. Differences in the assumed preferences of species between Armero and Cambao should be treated with caution given the vastly different sampling intensities between these two regions. *Megacephala cribrata* Steinheil was also reasonably abundant (16 individuals). *Calosoma alternans* contributed 62% of the total catch in Armero and 84% in Cambao. It dominated pastures in both localities.

### Carabid beetle assemblage structures

**Figure 2.**
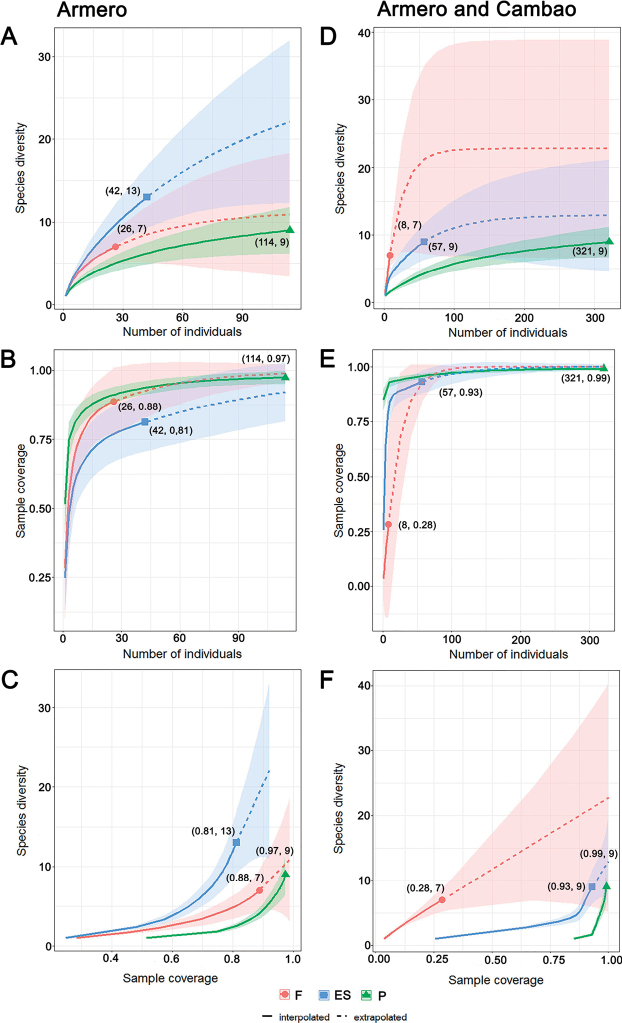
Rarefaction and extrapolation richness curves for carabid beetles from Armero (**A–C**), and Armero and Cambao combined (**D–F**) **A, D** comparison of richness between habitats using sample-size-based curves **B, E** sample completeness curves **C, F** comparison of richness using coverage-based curves. Abbreviations: F = forest, ES = early succession, P = pasture. Numbers in parentheses denote sample sizes and the observed Hill number (q = 0) (**A, D**), sample size and the estimated sample coverage (**B, E**), and the estimated sample coverage and the observed Hill number (q = 0) (**C, F**), respectively.

Sample size-based rarefaction/extrapolation curves showed no significant differences in species richness between habitat types in either datasets, as reflected by overlapping confidence intervals (Fig. 2). In Armero (13 months of data), early succession habitat appears to host more species than pasture and forest (Fig. 2A, C). Sample completeness (Fig. 2B) for all habitat types ranged between 81% and 97%, and estimated carabid species richness (Hill number q = 0) at 91% sample coverage were 9, 22, and 6 for forest, early succession, and pasture, respectively (Fig. 2C). Rarefaction/extrapolation curves for Armero and Cambao combined showed a different tendency, but with no significant difference between habitat types: forest had the highest number of species (Fig. 2D, F), but also had the lowest sample completeness (28%) (Fig. 2E), with an estimated richness of 22 (at 99% sample coverage), while early succession and pasture had 13 and 12 species, respectively (Fig. 2F).

**Figure 3.**
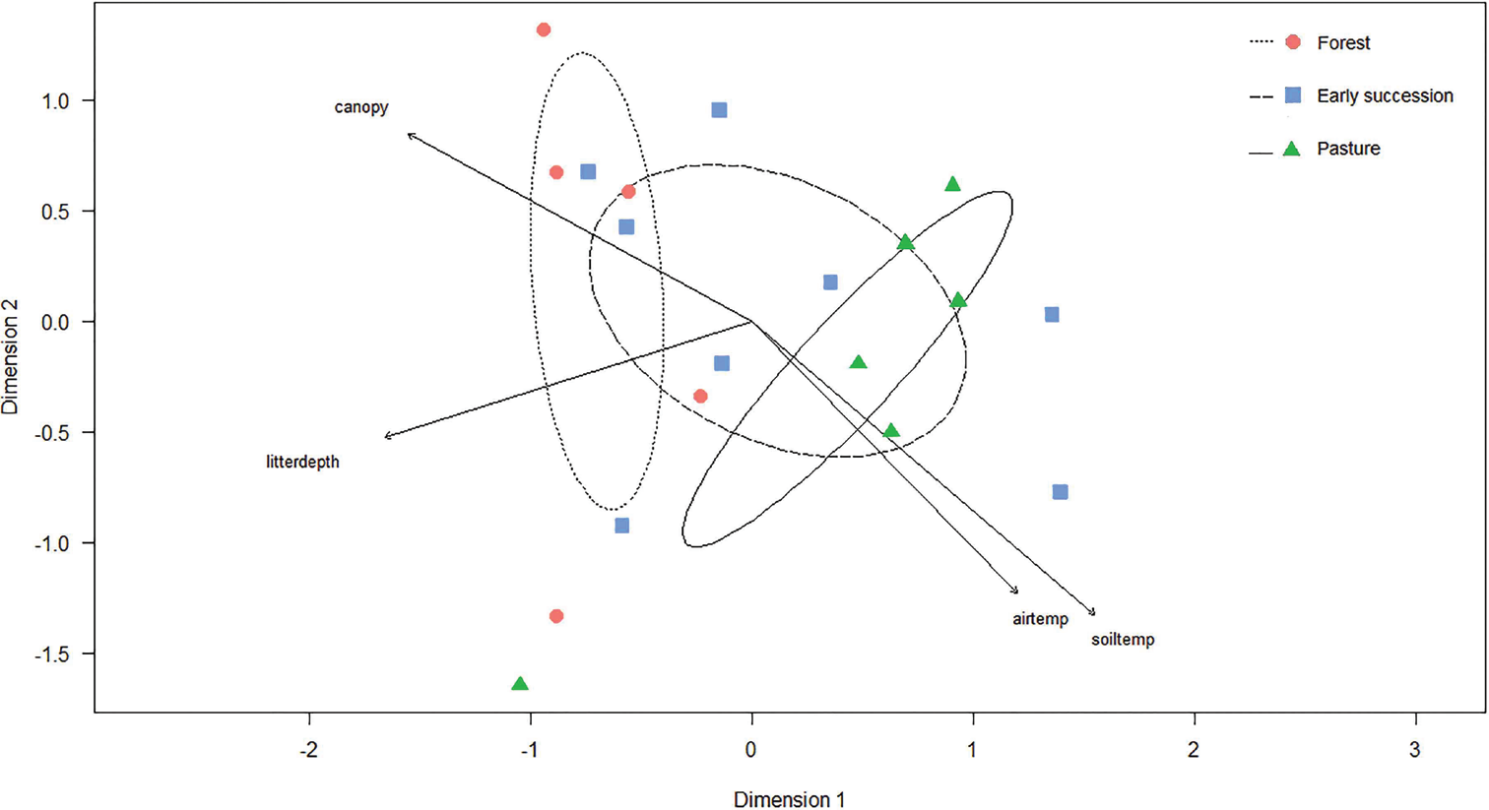
Non-metric multidimensional scaling ordination of carabid beetle assemblages at Armero (Colombia). Wet and dry season catches were analyzed and plotted separately. The catch in five of the ten forest samples returned zero individuals, and were removed from the analysis. The ellipses indicate 1 SD of the weighted average of site scores of forest (dotted line), early succession (long dashed line), and pasture (solid line). Abbreviations of the significant environmental vectors: soiltemp = soil temperature, airtemp = air temperature, litterdepth = leaf litter depth (cm), canopy = percentage canopy cover. Stress value 0.06.

The NMDS ordination for Armero showed that forest and pasture have characteristic and distinct species assemblages, while the early succession habitat type overlapped in assemblage structure with these other habitat types (Fig. 3). The assemblage in forest and pasture were most homogenous, while the early succession habitat was heterogeneous. Habitat type did not affect the carabid beetle assemblage significantly (PERMANOVA F = 1.281, p = 0.253), but the architectural variables like canopy cover (r^2^ = 0.342, p = 0.037) and leaf litter depth (r^2^ = 0.330, p = 0.041) did (Table 2). Although soil and air humidity did not influence the carabid beetle assemblage distribution significantly (r^2^ = 0.044, p = 0.694; r^2^ = 0.114, p = 0.380), soil and air temperature did (r^2^ = 0.452, p = 0.008; r^2^ = 0.321, p = 0.046), which related positively with the pasture beetle assemblage.

**Table 2.** Correlations (r^2^ and *p*-values) of vectors in the non-metric multidimensional scaling ordination of carabid beetle assemblages at Armero (Colombia).

	r²	*p*-value
Air humidity	0.114	0.380
Air temperature	0.321	**0.046**
Soil humidity	0.044	0.694
Soil temperature	0.452	**0.008**
Canopy cover	0.342	**0.037**
Leaf litter depth	0.330	**0.041**
Season	0.061	0.356

### Responses of individual species

**Figure 4.**
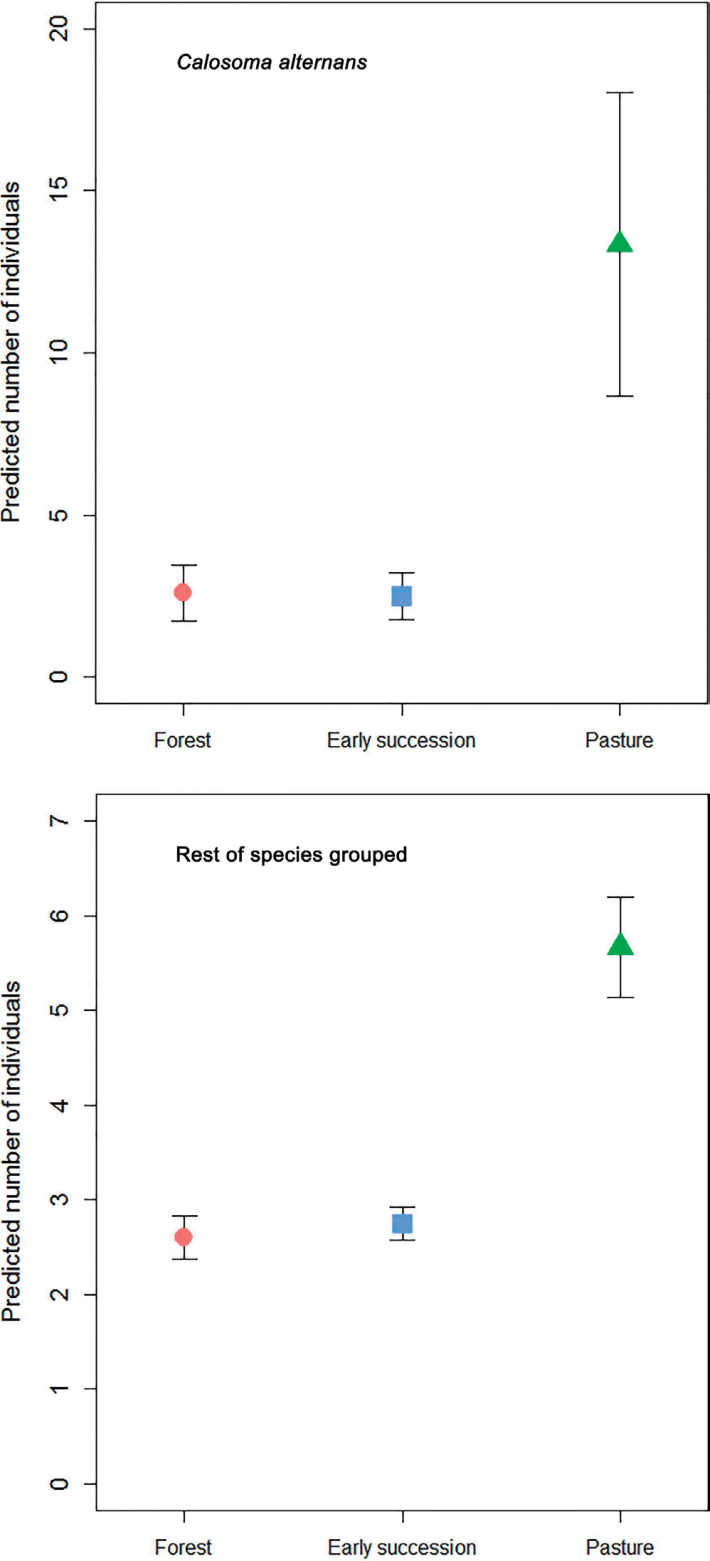
Generalized Linear Model predicted (mean ± SE) number of individuals of *Calosoma alternans* and the remaining carabid beetle species collected from Armero across the three habitat types (forest, early succession, and pasture). Note different y-axis scales.

**Figure 5.**
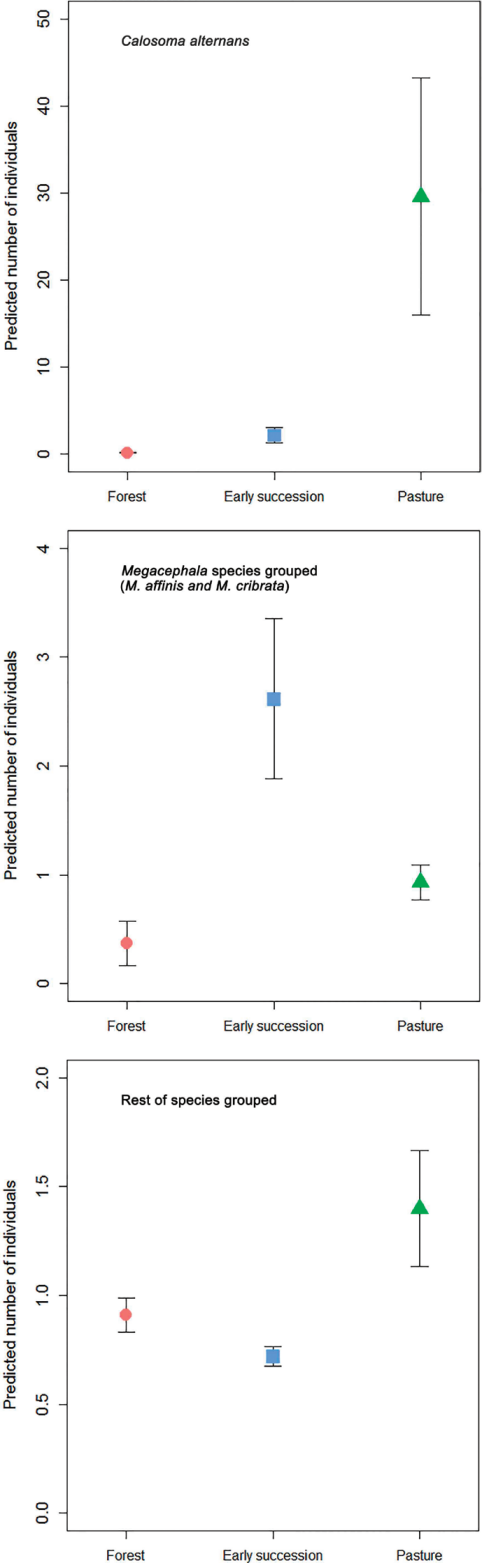
Generalized Linear Mixed Model predicted (mean ± SE) number of individuals of *Calosoma alternans*, genus *Megacephala* and the remaining carabid beetle species collected from Armero and Cambao combined across the three habitat types (forest, early succession, and pasture). Note different y-axis scales.

*Calosoma alternans* was most abundantly collected from pasture in both datasets (Table 3, Figs 4, 5): this habitat showed significant differences with both forest and early succession habitat types (Table 4). The same tendency was observed for the “rest of the species” group analyzed in Armero and Armero and Cambao combined, although without significant differences between habitat types. The combined Armero and Cambao dataset showed that the genus *Megacephala* (*M. affinis* and *M. cribrata*) was slightly most abundantly collected from early succession, with statistical differences between this habitat type and pasture (Table 4). None of the environmental variables were retained in the models (Table 3), except for litter depth (p = 0.001), which had a negative effect on *Megacephala* in the Armero and Cambao dataset.

**Table 3.** Generalized Linear Model and Generalized Linear Mixed Model results for carabid beetle species and species group (data of less abundant species pooled: Rest of the species) collected at Armero, and Armero and Cambao combined. Coefficients, standard errors (SE), and *p*-values are shown for intercepts, habitat type, season (wet and dry), and litter depth. Forest habitat type and dry season are in the intercept. Additionally, adjusted R^2^ and Mean Absolute Error (MAE) values from the k-fold cross-validation model performance procedure are shown.

		Intercept	Early succession	Pasture	Season	Litter depth	R²	MAE
**Armero**								
*Calosoma alternans*	Coefficient (SE)	-4.911 (0.984)	0.164 (0.887)	1.778 (0.744)	2.411 (0.761)		0.936	5.252
	p-value	**< 0.001**	0.855	**0.03**	**0.006**			
Rest of the species	Coefficient (SE)	-3.511 (0.408)	0.130 (0.441)	0.830 (0.41)	0.711 (0.311)		0.608	2.159
	p-value	**< 0.001**	0.771	0.061	**0.037**			
**Armero and Cambao**								
*Calosoma alternans*	Coefficient (SE)	-6.440 (1.396)	3.031 (1.323)	4.860 (1.293)	2.438 (0.577)		0.561	13.26
	p-value	**< 0.001**	**0.022**	**< 0.001**	**< 0.001**			
*Megacephala* species grouped	Coefficient (SE)	-3.169 (0.805)	1.218 (0.84)	-0.276 (0.899)		-1.688 (0.541)	0.737	1.174
(*M. affinis* and *M. cribrata*)	p-value	**< 0.001**	0.147	0.758		**0.001**		
Rest of the species	Coefficient (SE)	-2.424 (0.538)	-0.172 (0.665)	0.371 (0.629)			0.197	1.077
	p-value	**< 0.001**	0.796	0.555				

**Table 4.** Tukey’s HSD post‐hoc test results, comparing differences in the number of individuals of the carabid beetle species and species group collected in the three habitat types (forest, early succession, and pasture).

		Pasture – Forest	Early succession – Forest	Early succession – Pasture
**Armero**				
*Calosoma alternans*	Coefficient (SE)	1.778 (0.745)	0.164 (0.887)	-1.614 (0.622)
	p-value	**0.043**	0.981	**0.025**
Rest of the species	Coefficient (SE)	0.83 (0.41)	0.13 (0.441)	-0.7 (0.344)
	p-value	0.105	0.952	0.103
**Armero and Cambao**				
*Calosoma alternans*	Coefficient (SE)	4.86 (1.293)	3.031 (1.323)	-1.829 (0.587)
	p-value	**< 0.001**	0.052	**0.004**
*Megacephala* species grouped	Coefficient (SE)	-0.276 (0.898)	1.218 (0.84)	1.494 (0.476)
(*M. affinis* and *M. cribrata*)	p-value	0.947	0.303	**0.004**
Rest of the species	Coefficient (SE)	0.371 (0.629)	-0.172 (0.665)	-0.542 (0.53)
	p-value	0.825	0.964	0.56

### Seasonality

Seasonality did not affect the carabid beetle assemblage in Armero significantly (r^2^ = 0.061, p = 0.356) (Table 2), but did so for individual responses of *C. alternans* in both datasets (p = 0.006 in Armero, p < 0.001 in Armero and Cambao), and for the “rest of species” group (p = 0.037) in Armero (Table 3). Observed species richness was the same between the wet and dry season (14 species each season) in Armero, while Cambao had small differences (6 wet, 8 dry) (Table 1). However, abundances were higher during the wet season for both localities (78% for Armero and 90% for Cambao). These differences were mainly due to *C. alternans* being significantly more abundant during the wet season (Table 3). When *C. alternans* is removed, differences between seasons were smaller (66% of the catch during the wet season in Armero, and 62% in Cambao).

## Discussion

This study was performed during an El Niño event (2015/16), which means that the TDF ecosystem experienced harsh conditions, reflected by a considerable decrease in rainfall and an increase in air and soil temperatures (Montealegre 2014). We showed that the carabid beetle catches in both Armero and Cambao did not reach species saturation. Rarefaction/extrapolation curves approached an asymptote in pasture only, and pasture also showed a homogenous assemblage structure, distinct from forest. Early succession assemblage structure was highly heterogeneous, encompassing both forest and pasture, sharing 28% of species with both habitat types. Canopy cover, litter depth and soil and air temperatures were influential variables in structuring the carabid assemblage. Surprisingly, neither soil humidity nor seasonality affected assemblage structure significantly. Finally, *C. alternans* dominated pasture in both Armero and Cambao, while species of the genus *Megacephala* (*M. affinis* and *M. cribrata*) were more abundant in the early succession habitat type. None of the other species was collected abundantly enough to be analyzed individually.

The epigaeic carabid assemblage in tropical dry forest was species poor but with high dominance, like in other tropical carabid communities (Paarmann et al. 2002; Vieira et al. 2008; Rosero 2010). Only 12% of the species collected had more than 10 individuals, while 46% were singletons or doubletons. This low abundance was reflected in the rarefaction/extrapolation curves, which did not reach an asymptote, suggesting that epigaeic carabid beetle diversity in the TDF is certainly higher than presented here. Although rarity may be common in tropical ecosystems, the pattern we observed could appear as a consequence of an inappropriate sampling method and/or intensity (Magurran and Henderson 2011). Vennila and Rajagopal (1999) recommended more than 35 pitfall traps per site for quantitative studies in tropical agroforests, and Boetzl et al. (2018) showed that the use of guidance barriers could improve the efficiency of the catch. Furthermore, Liu et al. (2007) indicated that pitfall traps do not permit a complete inventory and recommended using light traps as a compliment. Many tropical carabid species live in the canopy (Erwin 1979), and will not be collected using pitfall trapping (see Kotze et al. 2011; Boetzl et al. 2018). To collect TDF carabid beetles more efficiently, we recommend increasing the number of traps and the length of the survey (one complete year of continuous trapping at minimum), use guidance barriers if possible, and include other methods to sample arboreal species. Additionally, high soil and air temperatures during the dry season (see Suppl. material 3: Table S3) result in the rapid evaporation of pitfall trap collecting fluid (Ariza 2016; pers. obs.), and we recommend using deeper pitfall traps with more collecting fluid. Apart from the method used here, the El Niño event, which coincided with our sampling, could be an important component to the depauperate carabid community in TDF. During a previous El Niño event (1996) in the Amazonian rainforest, carabid beetle richness decreased drastically compared with other periods (Lucky et al. 2002). In Mexican TDF, a decrease in Cantharidae beetle richness was also observed during the 1997/8 El Niño event (Pérez and Zaragoza 2016). Finally, the historic use of forest fragments and agricultural practices in pastures may explain the poor carabid community in this landscape. Harvey et al. (2008) indicated that beside severe fragmentation experienced in the TDF, contamination by agro-chemicals and illegal logging could be additional drivers of biodiversity loss. The degree to which these aspects affect carabid beetles in the region remains to be investigated.

The carabid beetle assemblage in the early succession habitat overlapped with assemblages in pasture and forest habitat types, a pattern not observed for dung beetles (Neves et al. 2010a) or arboreal ants (Neves et al. 2010b) in Brazilian TDF, where communities were more distinct between early succession and forest habitat types. Reasons for this discrepancy may be related to habitat complexity and the biology of these groups (Neves et al. 2010a, b). Dung beetles depend on ephemeral resources produced by larger animals that may be more sensitive to changes in the landscape (Hanski 1991), while ground-nesting ants in early successional TDF forage in trees, but do not do so in late successional stages (Neves et al. 2010b). For carabid beetles, early successional TDF sites may provide a mixed environment with elements from both pasture and forest, thus providing a heterogeneity of resources (Lebrija-Trejos et al. 2009) to be exploited by a subset of carabid beetle species. The lack of knowledge of the natural history of tropical species prevents us from identifying the habitat preferences of species found in the TDF early succession habitat type. Despite this, the little knowledge that do exist is reflected in the heterogeneous resource used by the carabid species collected from forest and early successional sites in Armero; *Apenes prasinus* Ball & Shpeley *Clivina* sp., and *Galerita* sp. are related to leaf-litter (Erwin 1991; Larochelle and Larivière 2003; Martínez 2005), while species shared between early succession and pasture (*Athrostictus paganus* (Dejean), *M. affinis* and *Tetragonoderus* sp.) prefer open areas and/or pasture (Larochelle and Larivière 2003; Vitolo 2004; Shpeley et al. 2017). *Calosoma alternans* (in Armero and Cambao) and *M. affinis* (Cambao) were present in all habitat types, suggesting that these species are habitat generalists. Indeed, *C. alternans* can be found in a wide variety of habitats and ecosystems (Gidaspow 1963), but also seems to prefer pastures (Bruschi 2010) as confirmed by our results. Nevertheless, it is difficult to attribute a particular habitat preference to species occurring in the early succession habitat type, partly because ground beetles readily disperse at the local level, even to suboptimal habitat (e.g., Niemelä and Halme 1992; Boetzl et al. 2016; Schneider et al. 2016; Knapp et al. 2019). Resources are heterogeneous and patchy, both at the fine and coarse scale (Wiens 1976; Pickett and Rogers 1997), thus carabid species of different preferences could occasionally occur in early successional stages, even if such habitat is suboptimal to them.

Soil and air temperatures were the only microclimatic variables that influenced the structuring of the carabid assemblage in our study. Carabid beetles, similar to other insects, are poikilothermic and sensitive to temperature (Beck 1983; Neven 2000; Bowler and Terblanche 2008), mainly during egg and larvae stages (Lövei and Sunderland 1996; Potter et al. 2009). Surprisingly, soil humidity did not affect the beetle assemblage, even though moisture is considered important in these dry forests (Balvanera and Aguirre 2006; Espinosa et al. 2011). During 2015/6, one of the strongest El Niño episodes occurred in Colombia (UNGRD 2016), producing a severe rainfall deficit that affected soil humidity negatively. This was reflected in our measurement of soil humidity, where differences between habitat and season were minimal. It is expected that in normal years, seasonality will result in more contrasting differences in soil humidity between open and forest habitats (Ceccon et al. 2006; Zhang et al. 2010). This could explain why variation in soil humidity did not affect carabid beetles significantly. Canopy cover and litter depth significantly influenced carabid assemblage structure in our study (see also Koivula et al. 1999; Antvogel and Bonn 2001; Taboada et al. 2008; Yu et al. 2008; Ogai and Kenta 2016). These structural variables can reflect and influence microclimatic conditions (Gardner 1991; Sanderson et al. 1995). In TDF, structural and environmental variables are related to succession but also depend on season, thus strong environmental gradients are not observed during the dry season (Lebrija-Trejos et al. 2009). For instance, TDF forests consists of deciduous trees that shed their leaves during the dry season, creating an open canopy (Murphy and Lugo 1986; Holbrook et al. 2009) thus minimizing differences between habitat types in terms of climatic gradients. On top of that, due to an extremely dry period because of the El Niño phenomenon (even during the wet season), microclimatic conditions (apart from soil and air temperature, see above) likely varied little between habitat types. Alternatively, conserving water is a challenger for small organisms in these harsh environments (Chown and Klok 2003), and as such, litter depth become an important environmental variable to these beetles in providing shelter (Koivula et al. 1999; Magura et al. 2005). Hopp et al. (2010) found that litter quantity was a better predictor of beetle assemblage recovery than soil humidity. Litter improve the environment in the soil (Facelli and Pickett 1991; Magura et al. 2004), offer habitat structure for organisms (Magura et al. 2000; Kalinkat et al. 2013) and supplies prey for carnivorous species (Guillemain et al. 1997).

Seasonality did not significantly influence the carabid beetle assemblage, even though numerous studies have shown seasonality to be important in dry forest beetles (e.g., Novais et al. 2016; Pérez and Zaragoza 2016; Rangel-Acosta and Martínez-Hernández 2017; Noguera et al. 2018). However, there are exceptions; for example dung beetle richness differences between wet and dry seasons in the Caatinga forest in Brazil were small (Medina and Lopes 2014). A reason for the lack of a seasonality signal in our data may, again, be due to a deficit in precipitation during the 2015/6 El Niño southern oscillation. Many carabid species diapause as an adaptation to harsh environmental conditions (Lövei and Sunderland 1996). Some observational studies have suggested that moisture could be a trigger for diapause development (Tauber et al. 1998; Hodek 2003). Either because of low humidity or environmental signals generated by the El Niño phenomenon, carabids species could display a lengthened diapause (during a drought event), thus resulting in the absence of seasonal peaks in their numbers (see Hanski 1987; Matsuo 2006). At the species level, *C. alternans* and the “rest of species” group did respond to the wet season. Some *Calosoma* species are associated with open habitats and dry soils (Larochelle and Larivière 2003), so a slight improvement of environmental conditions in the wet season during El Niño could be sufficient for this species to express seasonal peaks (e.g., Jacobs et al. 2011).

## Conclusions

Our study showed that early successional areas in TDF have a prominent role in the conservation of carabid beetles since it can act as a temporal habitat for a number of species that occur in forest and pasture. The loss of connectivity between dry forest patches limits the dispersal of species (Kindlmann and Burel 2008; Balzotti et al. 2020). Early successional stages could act as habitat corridors for carabid beetles, including some stenotopic species (e.g., Noordijk et al. 2008, 2009, 2011; Eggers et al. 2010), and promote the recolonization of forest patches. Land-use intensification homogenizes carabid assemblages (Meng et al. 2012), which is also the case for pasture in our study. In Armero and Cambao, pasture was dominated by *C. alternans*, which seems well-adapted to dry soils. We showed the importance of restoration to the recovery of this ecosystem. We demonstrated that climatic variation, like the El Niño episode, impacts the abundances and species richness of TDF carabid beetles markedly, necessitating the call for long-term studies to evaluate recovery in this landscape.

## Supplementary materials

Supplementary material 1 Table S1 Data type occurrences Explanation note List of plant species of each habitat type at Armero and Cambao. Abbreviations: F = forest, ES = early succession, P = pasture. https://binary.pensoft.net/file/555609This dataset is made available under the Open Database License (http://opendatacommons.org/licenses/odbl/1.0/). The Open Database License (ODbL) is a license agreement intended to allow users to freely share, modify, and use this Dataset while maintaining this same freedom for others, provided that the original source and author(s) are credited.Gloria Maria Ariza, Jorge Jácome, Héctor Eduardo Esquivel, D. Johan Kotze

Supplementary material 2 Table S2 Data type Linked Data Explanation note Information on the carabid species vouchers collected in Armero and Cambao and deposited in the Entomological Museum of the Universidad del Tolima, Colombia (MENT-UT). https://binary.pensoft.net/file/555610This dataset is made available under the Open Database License (http://opendatacommons.org/licenses/odbl/1.0/). The Open Database License (ODbL) is a license agreement intended to allow users to freely share, modify, and use this Dataset while maintaining this same freedom for others, provided that the original source and author(s) are credited.Gloria Maria Ariza, Jorge Jácome, Héctor Eduardo Esquivel, D. Johan Kotze

Supplementary material 3 Table S3 Data type environmental variables Explanation note Means (SD) of the environmental variables measured in Armero, Colombia during 13 months. The dry season period includes months 1–4, 7–9 and 13; wet season 5–6 and 10–12. For Cambao, data were collected one month during the dry and one month during the wet season. https://binary.pensoft.net/file/555611This dataset is made available under the Open Database License (http://opendatacommons.org/licenses/odbl/1.0/). The Open Database License (ODbL) is a license agreement intended to allow users to freely share, modify, and use this Dataset while maintaining this same freedom for others, provided that the original source and author(s) are credited.Gloria Maria Ariza, Jorge Jácome, Héctor Eduardo Esquivel, D. Johan Kotze
